# Development of label-free light-controlled gene expression technologies using mid-IR and terahertz light

**DOI:** 10.3389/fbioe.2024.1324757

**Published:** 2024-10-11

**Authors:** Hirohito Yamazaki, Ryusei Sugawara, Yurito Takayama

**Affiliations:** ^1^ Top Runner Incubation Center for Academia-Industry Fusion, Nagaoka University of Technology, Nagaoka, Japan; ^2^ Department of Mechanical Engineering, Nagaoka University of Technology, Nagaoka, Japan

**Keywords:** light-regulated technology, mid-infrared light, gene expression, label-free, photocage, photoswitch, terahertz light

## Abstract

Gene expression is a fundamental process that regulates diverse biological activities across all life stages. Given its vital role, there is an urgent need to develop innovative methodologies to effectively control gene expression. Light-controlled gene expression is considered a favorable approach because of its ability to provide precise spatiotemporal control. However, current light-controlled technologies rely on photosensitive molecular tags, making their practical use challenging. In this study, we review current technologies for light-controlled gene expression and propose the development of label-free light-controlled technologies using mid-infrared (mid-IR) and terahertz light.

## 1 Introduction

Light-controlled technologies have enabled researchers to control gene expression with spatiotemporal precision, leading to remarkable advancements in fundamental biological research and medical and pharmaceutical applications (Ryu et al., 2021; Jia and Sletten, 2022). This approach is ideal for studying biology because traditional methods that rely on chemical inducers or genetic modifications have inherent limitations in terms of specificity, reversibility, and control of the timing and location of gene expressions (Khalil, 2020; Ziegler et al., 2022). Photosensitive molecules have been modified into nucleotides, peptides, and small molecules to control *in vivo* and *in vitro* gene expression (Hoorens and Szymanski, 2018). Classically, ultraviolet (UV) light has been the main light source for activating photosensitive molecules because of its high energy and wide range. However, UV light exhibits shallow penetration in tissues and damages biological molecules, making it less ideal for biomedical applications (Sharma and Friedman, 2021). The recent development of visible light-reactive molecules has led to improvements in biocompatibility and expanded applications for cells (Dong et al., 2015; Weinstain et al., 2020). Moreover, much weaker light, such as near-infrared light (NIR), is promising for controlling biological activities due to less damage of biological molecules and better light penetration in tissues (Feng et al., 2021; Chen et al., 2020).

Although light-controlled gene expression-modulating technologies have become favorable for the regulation and study of biological systems, there are challenges. One of the challenges in conventional approaches is the requirement for the chemical conjugation of photosensitive molecules to target biological molecules. Specifically, researchers need to examine the target biological molecule structure and select suitable photosensitive molecules for the target process, as well as design a protocol for photosensitive molecule attachment that can yield a sufficient amount of the high-purity product. Another challenge is that the addition of photosensitive molecules for regulating gene expression using cell-free systems or living cells may interfere with enzyme activity, necessitating system optimization. For example, an appropriate laser power has to be chosen because a weak power may not trigger a photoreaction and a strong power can damage biological molecules, potentially leading to undesired photocatalysis. Therefore, optimization of parameters such as the chemical components and incubation conditions is necessary.

The main requirements of light-controlled gene expression within a completely natural and untreated system are a chemical component that can uniquely absorb the excitation light and that the light itself does not damage biological molecules. Considering these, a wavelength in the range of mid-infrared (mid-IR) to terahertz would be desirable because this range of light is absorbed in molecular vibration modes, which are specific to the molecules themselves. In this study, to aid the development of label-free light-controlled gene expression regulation systems, we review the potential of using mid-IR–terahertz wavelengths for regulating gene expression for fundamental research and practical applications. First, we discuss how light can interact with materials as the underlying concept in light-regulated technologies and introduce the common photosensitive molecule modification approaches, including photocages and photoswitches. Second, we outline the use of visible light and the NIR region for activating photosensitive DNA/RNA, proteins, and small molecules for regulating gene expression and discuss the challenges of the current light-controlled technologies, not at the life level, but at the basic conceptual level. Finally, we introduce the recently developed light-controlled technology utilizing mid-IR and terahertz wavelengths and address the challenges of this approach.

## 2 Photosensitive molecules: photocages and photoswitches

### 2.1 Role of light and photosensitive molecules in gene expression regulation

**FIGURE 1:**
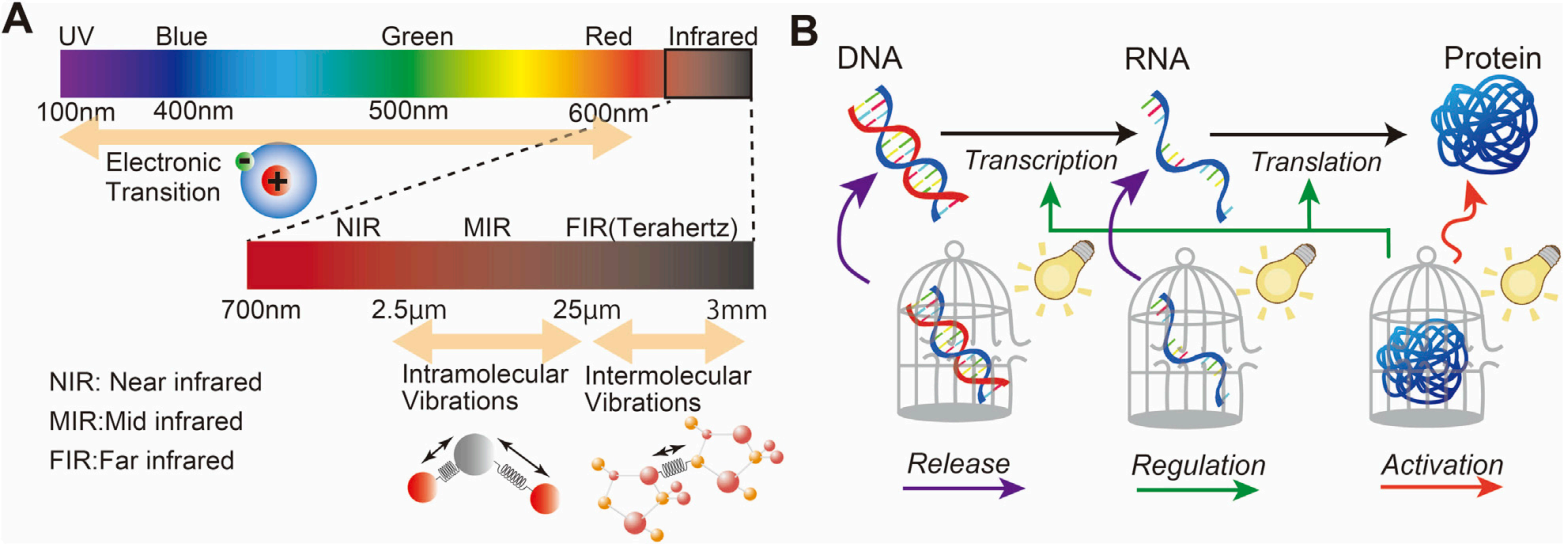
**(A)** Light wavelength for the excitation of electron transition, intramolecular vibrations, and intermolecular vibrations. **(B)** Overview of the basic biological process underlying gene expression and possible pathways for light-controlled gene expression.

Light is an electromagnetic wave that can interact with the molecules in the medium that it traverses (Gutzler et al., 2021). Light can be specifically made to interact with photoactive molecules to modulate gene expression (Hoorens and Szymanski, 2018). Upon the absorption of light, these molecules undergo specific photochemical reactions including cleavage and crosslinking, leading to chemical modifications, bond formation, and changes in their molecular structure (Tavakoli and Min, 2022). Light-controlled technologies provide a non-invasive approach to study and manipulate gene expression with high precision and selectivity. In this approach, a wide range of light can be applied, from UV to NIR and beyond. The photochemical reaction mechanism depends on the wavelength of light: UV and visible light (200–900 nm) leads to electronic transitions, while mid-IR/terahertz light (2.5 µm–3 mm) results in vibrational transitions, affecting intramolecular/intermolecular vibrations (Figure 1A). Two common strategies have been developed using photosensitive molecules: photocages and photoswitches. The following sections describe these strategies. For the ideal design of photosensitive molecules, the following characteristics are required (Singh et al., 2021): first, photosensitive molecules should show high light absorption at wavelengths that are not absorbed by or are not damaging to other biological molecules. In addition, the photoreaction should show high efficiency. Second, photosensitive molecules should have low intrinsic activity and should be stable in the reaction medium before light irradiation. Third, the by-products of the photoreaction should be transparent to the photoreactive trigger light to suppress other competitive reactions that may interfere with the designed photoreaction, and they should not react with molecules in the reaction medium. Furthermore, some photochemical reactions undergo several steps that involve excited/ground-state intermediates; therefore, the detailed mechanism of the photochemical reactions should be studied to understand the correct light irradiation.

Gene expression involves various biological processes, including transcription, where the DNA sequence of a gene is copied into complementary RNA, and translation, where messenger RNA (mRNA) serves as a template to assemble amino acids into polypeptide chains to form proteins. Proteins act as enzymes that modulate cellular metabolism and chemical reactions. Although light itself does not play an active role in this process, it can, with the assistance of photosensitive molecules, regulate a wide range of biological processes, including enzymatic activity. Figure 1B shows an example of this concept using photosensitive molecules of DNA, RNA, and proteins for light-controlled gene expression, which is further reviewed in Section 3.

### 2.2 Photocage

A photocage is a photoremovable protective molecule that blocks the bioactivity of the biological molecule to which it is conjugated. Photocage molecules absorb specific wavelengths of light, typically in the UV or visible range, and undergo a photoreaction that results in their removal or cleavage. This process releases the bioactive molecules and initiates their bioactivity. Popular photocages include boron–dipyrromethene (BODIPY), heptamethine cyanine (Cy7), and coumarin.

**FIGURE 2:**
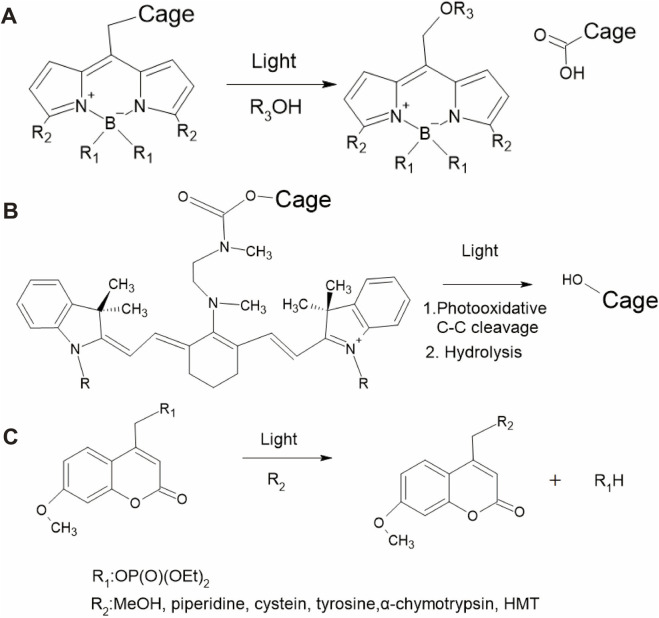
Reaction pathways of different photocage molecules. **(A)** BODIPY, **(B)** heptamethine cyanine (Cy7), and **(C)** 7-methoxycoumarin derivatives.

BODIPY is a fluorescence dye commonly used in bioimaging for biological studies and medical applications because it has low biotoxicity, is highly stable in various medium conditions, and has outstanding optical properties, such as high quantum yield, high absorption coefficient, narrow fluorescence spectrum, and long fluorescence lifetime (Singh et al., 2021). Additionally, structural modifications of BODIPYs can tune the absorption band to include the NIR region (Liu et al., 2019). However, the photolysis of some BODIPYs occasionally results in reduced photoreactivity (Singh et al., 2021), and real-time monitoring of the released active biomolecules is not feasible (Paul et al., 2019). Figure 2A shows an example of the photochemical reaction of BODIPY, where light irradiation initiates the cleavage of the cargo, resulting in a carbocation intermediate with a solvent-assisted nucleophilic attack (Peterson et al., 2018). A variety of BODIPY derivatives have been discussed in previous reviews (Singh et al., 2021; Cheng et al., 2023; Bobrov et al., 2021). Various types of caging groups can be used to create BODIPY photocages depending on the specific application and desired photorelease mechanisms. Some caging groups that are commonly attached to the BODIPY core include carboxylic acid, amine, alcohol, thiol, halide, xanthane, and phenol (Vohradská et al., 2018; Dockalova et al., 2020; Sitkowska et al., 2018).

Cy7 is another dye that is frequently used in bioimaging because it binds to biological molecules, including DNA, RNA, and proteins (Luciano et al., 2019; Rozovsky et al., 2019; Šmidlehner et al., 2018). The advantages of Cy7 are its low toxicity, low background absorbance interference, and high NIR light absorption (Yamamoto et al., 2019). Some Cy7 derivatives have been engineered to function *in vivo* by developing NIR-absorbing photocage molecules that are less harmful to tissues (Alachouzos et al., 2022; Janeková et al., 2022). The general photoreaction of the Cy7 photocage is shown in Figure 2B, where photooxidative cleavage at the C-C bond and hydrolysis result in the release of the caged molecule (Weinstain et al., 2020; Gorka et al., 2014).

Coumarins are widely used because they can be easily synthesized and rapidly released from substrates. As an example, the reaction of a photoactivatable phosphate-releasing group using 7-methoxycoumarin derivatives is shown in Figure 2C (Tavakoli and Min, 2022; Givens and Matuszewski, 1984). Using structural modifications, the photophysical properties of coumarins such as quantum yield and aqueous solubility can be improved (Bardhan and Deiters, 2019). The advantages of coumarin are its high absorption coefficient, high photoresponse efficiency, fast photolysis kinetics, and suitability for engineering two-photon experiments. Typically, coumarins can be connected to caging groups such as carbonates, alkoxides, carbamates, thiols, sulfonates, azides, halides, phosphates, and carboxylates (Weinstain et al., 2020).

### 2.3 Photoswitch

Photoswitches are photochromic molecules that undergo reversible conversion between two or more stable states under light irradiation. This photochemical isomerization process involves structural changes that result in distinct differences in the UV–visible absorption spectra of the isomeric states, making them photochromic. Mostly, photoswitch molecules show positive photochromism, with the generated species showing a higher maximum absorption (λ_max_) than the initial state (Bouas-Laurent and Dürr, 2001). However, when the initial molecules undergo bleaching upon photoisomerization, the photoswitch molecules show negative photochromism. Examples of such photoswitch molecules include azobenzenes, stilbenes, and spiropyrans.

**FIGURE 3:**
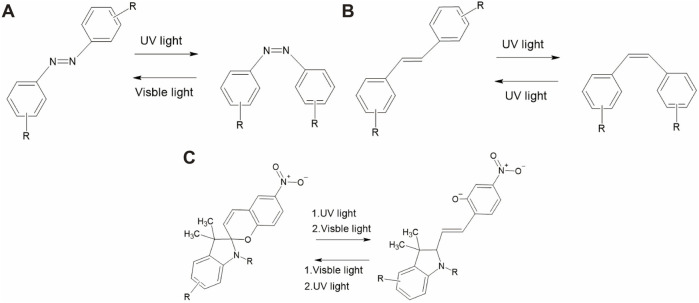
Reaction pathways of different photoswitch molecules. **(A)** Azobenzene, **(B)** stilbene, and **(C)** spiropyran.

Azobenzene is a well-studied photoswitch that exists in two distinct isomeric states: *trans* and *cis* (Axelrod et al., 2022; Volarić et al., 2022). They are widely used in biological applications because of their ease of synthesis, high quantum yield, low photobleaching, high photostationarity, and fast isomerization (Fuentes et al., 2020). Notably, *trans*-azobenzene is approximately 10 kcal/mol more stable than *cis*-azobenzene. The UV–visible absorption of *trans*-azobenzene exhibits two maximum peaks: a strong absorption peak near 320 nm due to a π–π* transition and a weaker peak absorption near 440 nm related to an n−π* transition (Gelabert et al., 2023; Kuntze et al., 2021). *cis*-azobenzene has a stronger absorption band of the π–π* transition near 400 nm and two shorter absorption bands at 280 and 250 nm (Dias et al., 1992). Absorption near 320 nm induces the rotation of the nitrogen double bond, leading to the formation of the non-polar *cis* isomer, whereas absorption at 440 nm is associated with *trans*-to-*cis* isomerization through various pathways (Giles et al., 2021). Figure 3A shows the structures of *trans*- and *cis*-azobenzene (Cattaneo and Persico, 1999). Thermal stimulation or visible light irradiation can induce a switch from the *cis* to *trans* isomer, which requires ∼95 kJ mol^−1^ of activation energy (Garcia-Amorós et al., 2018).

Stilbene is another photoswitch molecule that undergoes *trans–cis* isomerization (Villarón et al., 2021). It is a hydrocarbon comprising two phenyl rings connected by an ethylene (–CH=CH–) bridge that forms a central double bond. The photoisomerization mechanism of stilbene has been reported to be slightly different from that of azobenzene. The main reason is that the excited state of the *trans*-stilbene, initiated through the π−π* transition, is metastable (Han et al., 2002). Isomerization of unsubstituted stilbene occurs under irradiation near 300 nm, resulting in the formation of the *cis* isomer. An example of this transition is shown in Figure 3B. The thermal *cis*-to-*trans* reisomerization requires 41–46 kcal/mol, which cannot be achieved at room temperature. In terms of biological applications, the disadvantage of stilbene is its irreversible cyclization and oxidization to the *cis* isomer.

Spiropyran undergoes a photochromic reaction under UV irradiation, which induces heterocyclic cleavage at the C-O bond, leading to the formation of a zwitterionic structure, as shown in Figure 3C, an example of the transition pathway (Kortekaas and Browne, 2019). This isomerization process produces a significant polarity shift (8–15D) (Mukherjee et al., 2022; Shiraishi et al., 2023). More importantly, this process is reversible upon thermal stimulation and photochemical activation under visible light irradiation (>460 nm). The importance of spiropyran is that photoisomerization causes a significant change in polarity, which influences its hydrophilicity/hydrophobicity (Wang et al., 2021). Spiropyran has been reported to strongly interact with certain biological molecules (Keyvan Rad et al., 2022; Ali et al., 2019).

### 2.4 Challenges in using photosensitive molecules

**FIGURE 4:**
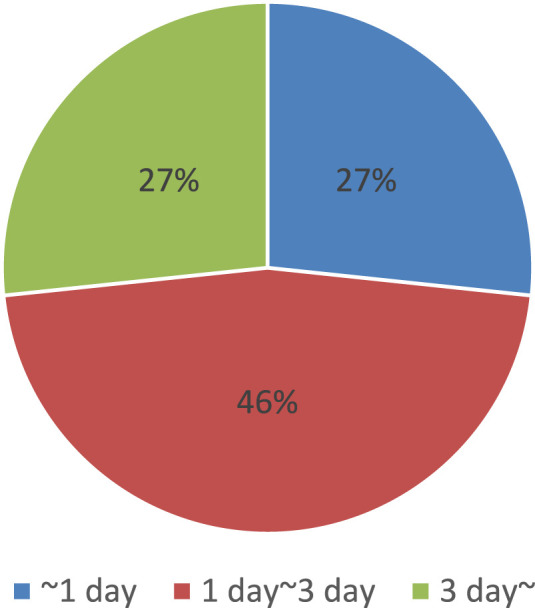
Summary of time required for photosensitive molecular modification, obtained from references (Becker et al., 2018; Cheong et al., 2024; Coelho et al., 2020; Conic et al., 2018; Erlendsson et al., 2019; Fields et al., 2019; Forero-Quintero et al., 2022; Gáspár et al., 2018; Horii et al., 2017; Kajimoto and Nakamura, 2018; Klena et al., 2023; Koch et al., 2021; Luo et al., 2023; Mai-Morente et al., 2021; Mazzucco et al., 2020; Mu et al., 2019; Munson and Ganley, 2016; Paweletz et al., 2022; Rawat and Sharma, 2024; Remenyi et al., 2019; Roberts et al., 2018; Sharma et al., 2019; Tanimizu, 2015; Teng et al., 2019; Tronnet and Oswald, 2018; Udupa et al., 2022; Wirth et al., 2020; Xu and Liu, 2019; York and Milush, 2015).

There are many candidates for photosensitive molecules that can be attached to target molecules. However, the challenge lies in the need to use specific attachment reactions, which limits the number of available pairs of photosensitive molecules and target molecules, or to implement tag modification. For example, prior to attachment, many photoswitch molecules are pre-labeled with protein tags such as SNAP tags, which can fuse with any target protein (Leng et al., 2017). Additionally, the modification process can be quite time-consuming. As an example, we analyzed the duration of photosensitive molecular modification based on protocol journal papers by picking up all related processes, as shown in Figure 4 (Becker et al., 2018; Cheong et al., 2024; Coelho et al., 2020; Conic et al., 2018; Erlendsson et al., 2019; Fields et al., 2019; Forero-Quintero et al., 2022; Gáspár et al., 2018; Horii et al., 2017; Kajimoto and Nakamura, 2018; Klena et al., 2023; Koch et al., 2021; Luo et al., 2023; Mai-Morente et al., 2021; Mazzucco et al., 2020; Mu et al., 2019; Munson and Ganley, 2016; Paweletz et al., 2022; Rawat and Sharma, 2024; Remenyi et al., 2019; Roberts et al., 2018; Sharma et al., 2019; Tanimizu, 2015; Teng et al., 2019; Tronnet and Oswald, 2018; Udupa et al., 2022; Wirth et al., 2020; Xu and Liu, 2019; York and Milush, 2015). The results indicate that the entire process, including preparation and related procedures, typically takes at least a day, with some cases requiring even more time.

Additionally, light absorption by photosensitive molecules is limited (Welleman et al., 2020). For practical use, sufficient light penetration through the cells and tissues without causing damage is ideal. Nevertheless, most photosensitive molecules have not yet been engineered to operate at longer wavelengths, such as NIR light (Jia and Sletten, 2022).

Furthermore, some photosensitive molecules show inherent poor water solubility, which might cause biomolecular aggregation and result in interference with gene expression (Deng et al., 2021; Poryvai et al., 2022; Berdnikova, 2019). For instance, traditional BODIPY dyes dissolve only in organic solvents, which is why, to date, tremendous efforts have been made to improve their water solubility by introducing hydrophilic groups such as phosphonates, quaternary ammonium salts, and sulfonates (Koh et al., 2019; Mao et al., 2020; Zhou et al., 2020).

Finally, the development of a new photosensitive molecule design is likely serendipitous (Deng et al., 2021; Dcona et al., 2020). Therefore, laborious efforts are often required to develop photosensitive molecules that are suitable for any untried gene expression system of interest (Buglioni et al., 2022; Roßmann et al., 2023).

## 3 Current light-controlled gene expression technology

In this section, we review how light irradiation can be used to control gene expression. As shown in Figure 1B, light irradiation can activate photosensitive DNA/RNA, proteins, and small molecules that influence gene expression; these categories are discussed in the following sections.

### 3.1 DNA/RNA-based light-controlled gene expression

The photocontrol of gene expression using nucleic acids is typically facilitated by photosensitive molecule-modified DNA or RNA, as shown in Figure 1B. There are two main strategies for this approach: light-induced gene activation and light-induced knockdown using photocage molecule-modified oligonucleotides.

**FIGURE 5:**
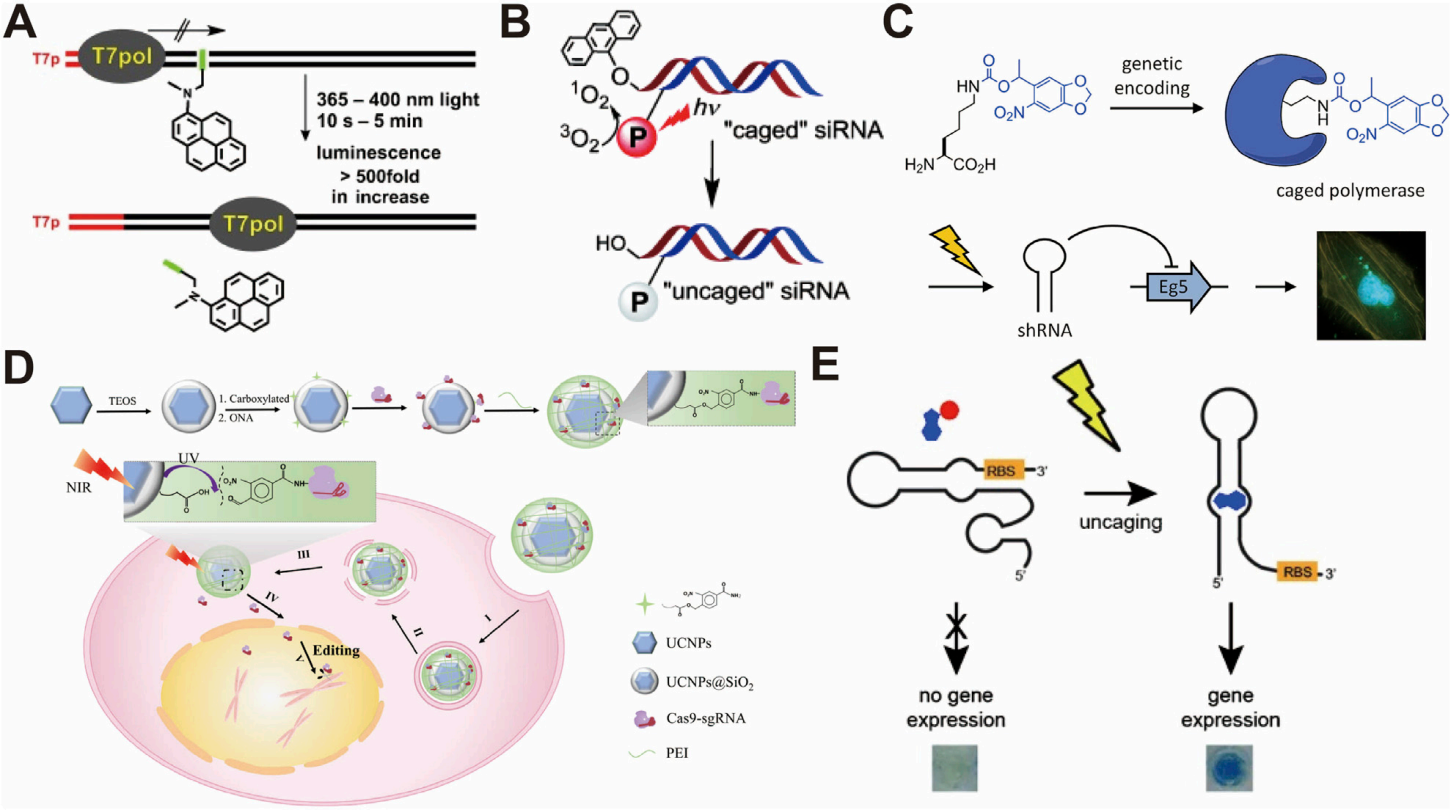
**(A)** Selective activation of psoralen-cross-linked DNA with UV and blue light (Stafforst and Stadler, 2013). **(B)** Photoactivation of siRNAs with red light for a non-toxic cellular approach (Meyer and Mokhir, 2014). **(C)** Spatiotemporal control of gene expression using photocaged T7 RNA polymerase (Hemphill et al., 2013). **(D)** Light-responsive nanocarrier for precise CRISPR-Cas9-mediated gene editing (Pan et al., 2019). **(E)** Spatiotemporal control of gene expression using riboswitches and photocaged ligands (Walsh et al., 2014). All figures have been adapted with permission from John Wiley and Sons Copyright (2013) and (2014), American Chemical Society Copyright (2013), and Springer Nature Copyright (2019).

Active light control of transcription and translation has been demonstrated by modifying oligonucleotides with photocage molecules. The modifications in the oligonucleotides can be introduced into the phosphate backbone, Watson–Crick face, or nucleotide base. For example, gene expression in cells and synthetic cells has been controlled using photolabile groups on the phosphate backbone of the DNA and RNA modified with photosensitive molecules, including coumarin (Kaufmann et al., 2023; Ando et al., 2001), 2-nitroveratryl bromide (Hartmann and Booth, 2023), and thioether-enol phosphate (Feng et al., 2017; Wang et al., 2016). Other than at the DNA backbone, psoralen cross-linking at the Watson–Crick face of the DNA promoter impedes the unwinding of the double helix, resulting in the blocking of the transcription process (Figure 5A) (Stafforst and Stadler, 2013; Stadler and Stafforst, 2014). Photosensitive molecules can also be introduced at nucleotide bases. Nucleotides modified with photosensitive molecules, such as benzophenones (Anhäuser et al., 2020), diethylaminocoumarin (Menge and Heckel, 2011), 2-nitrobenzyl bromide (Chakrapani et al., 2020), and 6-nitropiperonyloxymethyl group (Lee et al., 2021), have been integrated into DNA or RNA templates for light-controlled transcription and translation. When photoswitch molecules are modified into DNA/RNA, they can reversibly regulate the transcription and translation processes based on light irradiation. For instance, photoswitch molecules, including azobenzene (Tian et al., 2016; Xing et al., 2015; Dudek et al., 2018), stilbenes (O’Hagan et al., 2019; O’Hagan et al., 2020), arylstilbazolium (Czerwinska and Juskowiak, 2012), and 8-pyrenylvinyl deoxyguanosine (Ogasawara, 2018), which contain G-quadruplexes in a hyperstable state, can effectively inhibit transcription and translation processes. Light irradiation induces an unstable state, allowing activity, and additional light irradiation in the unstable state recovers hyperstable G-quadruplexes.

One approach to knock down gene expression is to use photocage-modified antisense oligonucleotides (ASOs), which are short single-stranded nucleotides that specifically bind to target DNA or RNA to stop transcription and translation. Modification of ASOs with 2-nitrobenzyl-caged thymidines has been shown to cause the photoactive knockdown of cancer-related genes (Govan et al., 2013; Tang et al., 2007; Sakamoto et al., 2014) and developmental genes (Tallafuss et al., 2012; Deiters et al., 2010). The inclusion of 2-nitrobenzyl can be achieved in the base-pairing region, nucleobase, and backbone of the complementary strand. Circularized ASOs can also be formed with photocleavable linkers, such as coumarin- (Yamazoe et al., 2014), 2-nitrobenzyl- (Yang et al., 2018), quinoline- (O’Connor et al., 2019), and Ru-BEP (Griepenburg et al., 2015), which allows the control of gene expression using light irradiation. Another approach is to use small interfering RNAs (siRNAs), which are approximately 20 base pairs of dsRNAs, that specifically suppress gene expression by destroying mRNA. By attaching 2-nitrobenzyl on the phosphate backbone and 5′ or 3′phosphate termini of siRNAs, light-controlled knockdown could be achieved in zebrafish (Blidner et al., 2008), mammalian cells (Zhang L. et al., 2018), and HeLa cells (Yu et al., 2018; Kala et al., 2014). When a 9-alkoxyanthracenyl fragment as an O_2_-sensitive moiety is incorporated in photocage molecules, the selectivity of the activation wavelength can be tuned by attaching photosensitizers to the 3′-terminus (Figure 5B) (Meyer and Mokhir, 2014). The use of light-responsive nanoparticles (NPs) is also a common approach for spatiotemporally manipulating siRNA functions. Typically, irradiation with NIR light triggers the release of thiol-modified siRNAs attached to gold NPs, resulting in the NIR-controlled knockdown of gene expression in cells (Huang et al., 2015; Riley et al., 2018; Braun et al., 2009). In addition, siRNA/mPEG-b-P(APNBMA) NPs containing o-nitrobenzyl moieties have been engineered to initiate siRNA release post-UV light irradiation (Foster et al., 2015).

### 3.2 Protein-based light-controlled gene expression

Photocontrol of proteins can affect the regulation of DNA/RNA synthesis or activity and the direct activation of protein function, as shown in Figure 1B, using photosensitive molecule-modified proteins.

The specific incorporation of amino acids with photosensitive molecules is required to generate photosensitive molecule-modified proteins in cells (Arbely et al., 2012; Chou and Deiters, 2011). One approach to demonstrate this incorporation is to select an amber stop codon as the insertion site for amino acids with photosensitive molecules (Wang et al., 2012; Neumann, 2012). Examples of this approach include the modification of a nitrobenzyl group on RNA polymerases to allow light-controlled gene expression in cells, as shown in Figure 5C (Hemphill et al., 2013; Chou et al., 2010). Additionally, other light-activated gene-editing tools, such as Cre recombinases (Edwards et al., 2009) and zinc finger (ZF) nucleases (Chou and Deiters, 2011), have been engineered by modifying o-nitrobenzyl on essential residues in catalytic sites, enabling activation upon light irradiation (Chou and Deiters, 2011; Luo et al., 2016). By fusing the GIGANTEA (GI) protein with the ZF protein, blue light irradiation induces heterodimerization between the GI and the light–oxygen–voltage (LOV) domain, which assists the transcriptional activation domain VP16 in initiating the transcription of the gene of interest (Polstein and Gersbach, 2012). Similarly, when the light-sensitive cryptochrome 2 (CRY2) protein is fused with transcription activator-like effector (TALE) proteins, blue light-induced conformational changes in CRY2 recruit a CIB1–effector domain, which exerts the active control of transcription in the same endogenous genome (Konermann et al., 2013). Furthermore, 2-nitrobenzyl-modified tamoxifen is used in light-controlled Cre systems to effectively regulate gene expression in cells (Inlay et al., 2013). Optical dimerization of the CRY2–CIB system has been widely used to reconstitute the split recombinase of Cre and Flp, which enables gene expression control in cells (Cautereels et al., 2024) and mouse brains (Jung et al., 2019).

The CRISPR-Cas9 system is also a well-known gene editing tool that can delete, replace, or insert any part of the genome sequence and has been extensively explored for the light-activated control of gene expression. Light-activated control of the CRISPR-Cas9 system has been achieved by engineering light-responsive Cas9 (Hemphill et al., 2015), gRNA (Moroz-Omori et al., 2020; Jain et al., 2016), and transcription factors (Shao et al., 2018). An example of an engineered Cas9 nuclease is the incorporation of a 2-nitrobenzyl-modified lysine amino acid, which reversibly affects gene function (Hemphill et al., 2015). As shown in Figure 5D, when Cas9 is bound to lanthanide-doped upconversion NPs by a 2-nitrobenzyl photocage, NIR irradiation produces local UV light, resulting in a photocleavage reaction and the release of the CRISPR-Cas9 system (Pan et al., 2019). Other candidate proteins, such as light-activated phosphorylation (Nguyen et al., 2018), dimeric green fluorescent protein (Zhou et al., 2018), and cyclic diguanylate monophosphate signaling cascades (Shao et al., 2018), have also been reported for use in light-activated CRISPR-Cas9 systems.

### 3.3 Small-molecule-based light-controlled gene expression

In addition to DNA, RNA, and proteins, small-molecule compounds are also general candidates for the photoreactive control of gene expression. Popular small molecules include nucleotides, peptides, and ligands. Nucleotides are the basic building blocks that constitute DNA and RNA; thus, the photocontrol of nucleotides is a straightforward approach for controlling gene expression. In this regard, [7-(diethylamino)coumarin-4-yl]methyl (DEACM)-modified ATP and 2-nitrobenzyl-modified UTP/GTP have been demonstrated as molecules to photocontrol the transcription reaction *in vitro* (Shao et al., 2018; Pinheiro et al., 2008). The modification of ligands with 2-nitrobenzyl and azobenzene, which effectively interact with DNA/RNA, is a useful approach for controlling gene expression (Walsh et al., 2014; Young et al., 2009; Paul et al., 2021). An example molecule is theophylline, which can specifically bind to the mRNA riboswitch; the photocontrol of gene expression could be achieved by modifying theophylline with 2-nitrobenzyl (Figure 5E) (Walsh et al., 2014). The binding affinity of tamoxifen and cyclophen to the estrogen receptor (ER)-binding domain can be utilized for photoactive regulation by modifying tamoxifen and cyclophen with 2-nitrobenzyl, coumarin, and cyanine derivatives (Zhang W. et al., 2018; Wong et al., 2017; Cruz et al., 2000). The tetracycline (Tet) system in mammalian cells is controlled by nitrobenzyl-modified doxycyclines (Cambridge et al., 2006). Many other small molecules, such as coumarin-modified cAMP-response element-binding protein (CREB) inhibitor (Imoto et al., 2020) and 2-nitrobenzyl-modified ecdysone and nuclear hormones (Link et al., 2004; Lin et al., 2002), have also been engineered to regulate gene expression in cells.

### 3.4 Challenges with current light-controlled gene expression approaches

To understand the nature of cells, photosensitive molecules can be used to probe fundamental aspects, such as molecular reactions, molecular kinetics, and live cell dynamics. However, the introduction of photosensitive molecules into cells or cell-free systems might influence molecular activity, thereby biasing the outcomes of the study. For example, exogenous photosensitive molecules in live cells can potentially harm normal cellular activities, including unintended effects on cell behavior, signaling pathways, and cellular homeostasis (Chen et al., 2022). Some photosensitive molecules may exhibit cytotoxic effects that affect cell viability and overall cellular health (Dcona et al., 2020). Therefore, researchers must carefully assess the concentration and exposure time required to minimize cytotoxicity.

**FIGURE 6:**
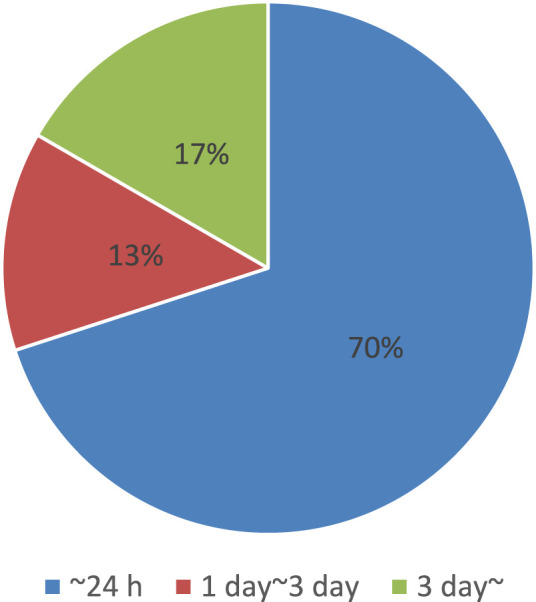
Summary of time required for electroporation, obtained from references (Alfastsen et al., 2021; Biase and Schettini, 2024; Chan et al., 2023; Clements et al., 2024; Crestani et al., 2022; Denoth-Lippuner et al., 2022; Egashira et al., 2023; Flumens et al., 2023; Gao et al., 2022; Gesuita et al., 2022; Heinz et al., 2020; Lankford and Hulleman, 2024; Laprie-Sentenac et al., 2023; Lax et al., 2022; Layden et al., 2021; Li et al., 2023; Ling et al., 2022; Meka et al., 2024; Nagahama et al., 2021; Nomura et al., 2020; Sakamoto et al., 2022; Sanketi et al., 2023; Skruber et al., 2021; Turchetto et al., 2020; Wang et al., 2020; Williams and Sauka-Spengler, 2021; Wilson et al., 2020; Wu et al., 2023; Xu et al., 2021; Xu et al., 2020; Zhou et al., 2021).

As mentioned in Section 2.4, the introduction of photosensitive molecules is also a lengthy process. As shown in Figure 6, which summarizes the total time required for molecular introduction into cells using electroporation based on protocol journal papers by picking up all related processes, the process can be done within a day. However, in certain cases, it can take a significantly longer time (Alfastsen et al., 2021; Biase and Schettini, 2024; Chan et al., 2023; Clements et al., 2024; Crestani et al., 2022; Denoth-Lippuner et al., 2022; Egashira et al., 2023; Flumens et al., 2023; Gao et al., 2022; Gesuita et al., 2022; Heinz et al., 2020; Lankford and Hulleman, 2024; Laprie-Sentenac et al., 2023; Lax et al., 2022; Layden et al., 2021; Li et al., 2023; Ling et al., 2022; Meka et al., 2024; Nagahama et al., 2021; Nomura et al., 2020; Sakamoto et al., 2022; Sanketi et al., 2023; Skruber et al., 2021; Turchetto et al., 2020; Wang et al., 2020; Williams and Sauka-Spengler, 2021; Wilson et al., 2020; Wu et al., 2023; Xu et al., 2021; Xu et al., 2020; Zhou et al., 2021).

Furthermore, achieving high specificity and selectivity for target molecules can be challenging. Ideally, photosensitive molecules should interact only with the intended targets; however, off-target effects may occur, leading to the misinterpretation of the experimental results (Laczi et al., 2022). Moreover, efficient delivery of light-sensitive molecule-tagged biomolecules to specific locations in live cells is required to achieve the desired control of gene expression (Deng et al., 2020).

In addition, light irradiation can induce cellular stress and damage, which is known as phototoxicity. This is particularly relevant in live-cell imaging studies, in which prolonged exposure to light may lead to alterations in cellular behavior (Talone et al., 2021). Different cell types respond differently to photosensitive molecules (Powell et al., 2018). It is important to consider the specific characteristics of the cell type under investigation and validate the applicability of the chosen photosensitive molecule to the cell system of interest. The above discussion highlights that the development of light-regulated translation technologies requires addressing many challenges through enormous efforts, testing, and optimization to improve the effectiveness of the methodologies (Welleman et al., 2020).

## 4 Use of mid-IR–terahertz light for label-free light-controlled gene expression

Based on the challenges discussed above, we propose that mid-IR and terahertz light can be used as solutions for light-controlled gene expression regulation; this section discusses this proposal in detail.

### 4.1 Potential for using mid-IR–terahertz light for gene expression regulation

In principle, as described in Section 2.1, mid-IR and terahertz light (2.5 μm–3 mm) has a strong interaction with intramolecular/intermolecular vibrations (López-Lorente et al., 2016; Tonouchi, 2007; Ma et al., 2019; Hoshina et al., 2020). Therefore, mid-IR and terahertz light has been used as light sources for imaging with high special selectivity and sensitivity and molecular detection (Jain et al., 2020). For example, mid-IR photothermal imaging is an emerging technology that can effectively excite the target vibration modes in biological molecules. This results in local property changes, such as the refractive index, molecular volume, and vibration modes, and monitoring these changes allows a high spatial resolution with low photodamage to biological molecules (Samolis et al., 2023; Ishigane et al., 2023).

**FIGURE 7:**
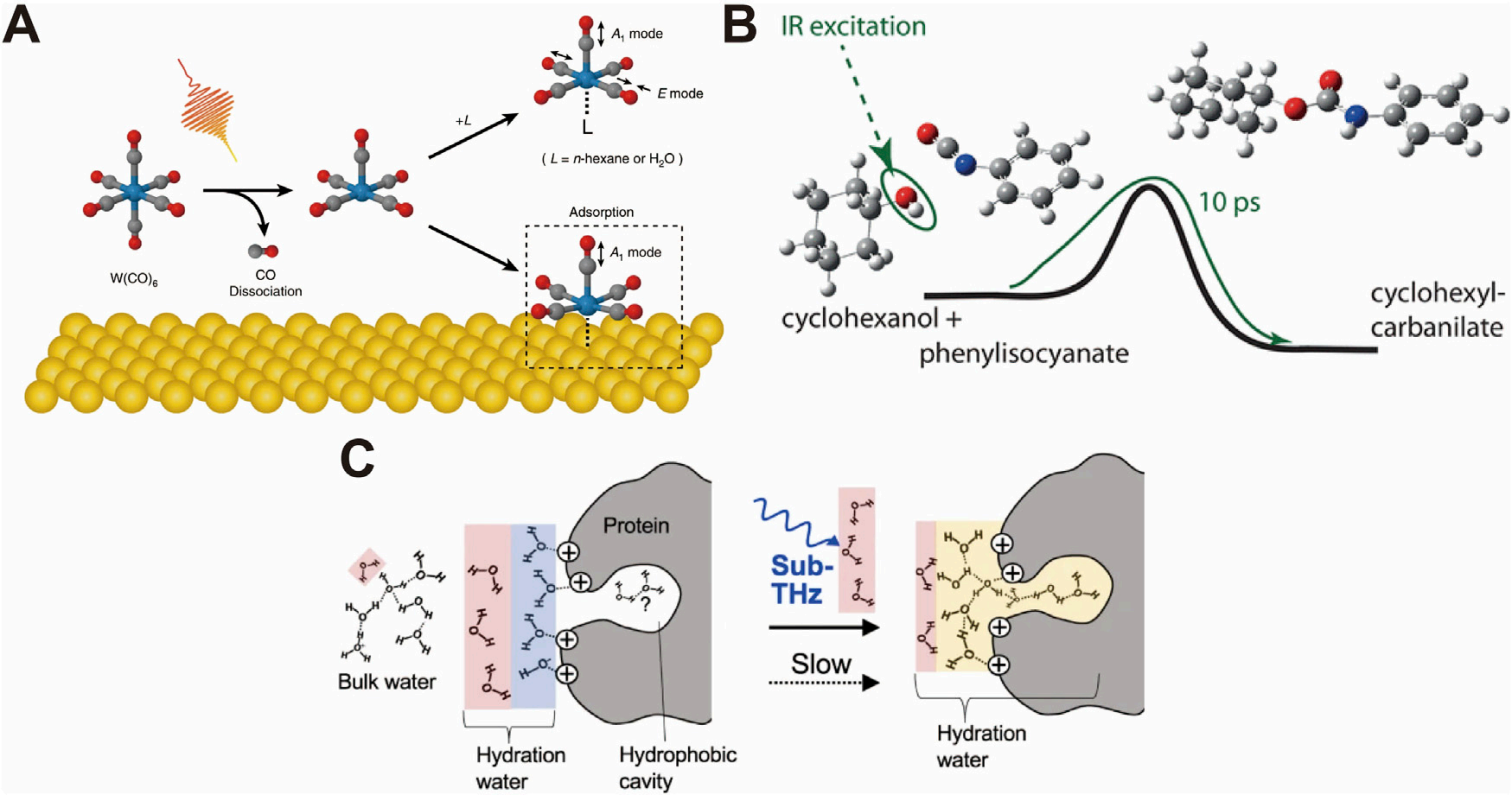
**(A)** Plasmonic enhanced mid-IR light field dissociation of CO of n-hexane (Morichika et al., 2019). **(B)** Selective vibrational excitation-induced ground-state reactions by mid-IR light (Heyne and Kühn, 2019). **(C)** Terahertz light promoting the hydrophobic hydration around the protein (Sugiyama et al., 2023). All figures have been adapted with permission from American Chemical Society Copyright (2019) and Springer Nature Copyright (2019), (2023).

Since mid-IR and terahertz light can interact with specific molecular vibrations, the irradiation of these light sources can influence biological molecular activity; thus, there is potential to control specific gene expression. The strong light energy at this wavelength can influence molecular activity in a label-free manner (Morichika and Ashihara, 2022; Park et al., 2018; Windhorn et al., 2002). However, to the best of our knowledge, mid-IR and terahertz light has not yet been commonly used for the light control of gene expression. One of the reasons for this is the high light absorption of water in this wavelength range, which leads to an increase in ambient temperature and hampers biomolecular activity (Holmstrom and Nesbitt, 2010; Ashwood et al., 2020). A short-pulse laser can break this fundamental barrier because cyclical ultrashort-term heating and cooling suppress the increase in ambient temperature (Toda et al., 2019; Zhang et al., 2019). Interestingly, when analyzing the energy of mid-IR and terahertz light (2.5 µm–3 mm), which corresponds to 7.95 × 10^−20^ to 6.62 × 10^−23^ joule/photon, the energy of biological processes, such as hydrolysis of a peptide bond (−1.39 × 10^−20^ to −2.78 × 10^−20^ joule/molecule), ATP hydrolysis (−5.21 × 10^−20^ joule/molecule), and folding energies of RNA secondary structures (−2.78 × 10^−20^ to −1.11 × 10^−19^ joule/molecule), is not very different (Jia et al., 2021). In Figure 7A, Morichika et al. (2019) showed that the irradiation of a plasmonic structure using a mid-IR pulse laser enhanced the localized light field, resulting in CO dissociation of n-hexane mediated by vibrational ladder climbing (Morichika et al., 2019). Other plasmonic studies have showed that strong mid-IR light in a plasmon nanocavity perturbs a few-nm-thick shell of water, predicting the mid-IR and terahertz light interaction with the molecular reaction (Chikkaraddy et al., 2022). Even without plasmonic enhancement, pulsed mid-IR light can selectively induce vibrational excitation, allowing the activation energy barriers to be overcome and facilitating ground-state reactions with minimal heat generation in Figure 7B (Stensitzki et al., 2018). This can result in bimolecular alcoholysis reactions, bidirectional tautomerization of thiotropolone, and the formation of urethane and polyurethane (Stensitzki et al., 2018; Heyne and Kühn, 2019; Nunes et al., 2020).

Although understanding the detailed mechanism of mid-IR and terahertz light irradiation on live cells is complicated, Toyama et al. (2022) showed that strong pulsed-light irradiation of *E. coli* cells in the mid-IR absorption region can affect their growth rate and survival. Furthermore, the much weaker energy of terahertz light influences the biological molecular activities. Recently, Sugiyama et al. (2023) discovered that the irradiation of pulsed terahertz light on proteins enhances hydrophobic hydration, leading to an increase in the number of hydrogen bonds at the hydration layer in Figure 7C, suggesting that even terahertz light can influence gene expression (Sugiyama et al., 2023). Although previous reports did not show clear findings, some previous studies have already predicted the influence of protein activity and expression (Sun et al., 2021). For example, Tan et al*.* found the downregulation of SYN expression in primary hippocampal neurons and PSD95 expression in cortical neurons under 0.16–0.17-THz irradiation, with a positive correlation with the exposure time and laser power (Zhi et al., 2019).

### 4.2 Challenges associated with mid-IR–terahertz light technology

Although some previous studies have shown the effect of mid-IR and terahertz light at the molecular level and presented some molecular reactions, many challenges remain for the active utilization of these wavelengths of light. First, the vibrational mode of molecules is influenced by a variety of factors, such as temperature, humidity, and substrates. Thus, an absorption spectrum shift of target biological molecules can occur in the environment, and researchers may need to calibrate the excitation wavelength. Second, as mentioned above, water absorption of mid-IR and terahertz light is quite high (100–10^4^ cm^−1^); therefore, limited light penetration might be a critical issue when target samples are a large volume of biological molecules or tissues (Prahl, 2017). Third, instruments and optical elements for the mid-IR and terahertz regions are often expensive and not widely available for commercial purchase, necessitating researchers to develop a setup if cost considerations come into play. Fourth, targeting specific molecules is required to obtain absorption spectrum profiles using Fourier-transform infrared spectroscopy, which requires the preparation of a large volume of purified biological samples.

## 5 Conclusion

Here, we provide an overview of light-controlled gene expression and propose a label-free light control approach using mid-IR and terahertz light. First, we explain the interaction between light and the materials, offering insights into the selection of appropriate wavelengths. Second, we introduce common techniques for modifying photosensitive molecules, such as photocages and photoswitches, and review previous studies on light-controlled gene expression via the manipulation of photosensitive DNA/RNA, proteins, and small molecules. In addition, we discuss the technological challenges associated with the current technologies utilizing photosensitive molecules for the regulation of gene expression. Looking ahead, we explore the exciting prospect of extending label-free light-controlled technologies using the mid-IR and terahertz wavelength ranges, which are traditionally limited by water absorption, in biological applications. Because it allows the photocontrol of gene expression without any photosensitive molecular tag, this uncharted territory shows potential for groundbreaking innovations in the field of gene expression, contributing to medical and pharmaceutical development.
